# Urgent care study of the LumiraDx SARS-CoV-2 Ag Test for rapid diagnosis of COVID-19

**DOI:** 10.1186/s41512-021-00113-7

**Published:** 2021-12-24

**Authors:** Jared Gresh, Harold Kisner, Brian DuChateau

**Affiliations:** 1 Compass Medical, East Bridgewater, MA USA; 2 LumiraDx, Waltham, MA USA; 3 NECAPS INC, Charlestown, RI USA

**Keywords:** Antigen testing, Community-based setting, COVID-19, False negative, SARS-CoV-2, Rapid diagnosis

## Abstract

**Background:**

Testing individuals suspected of severe acute respiratory syndrome–like coronavirus 2 (SARS-CoV-2) infection is essential to reduce the spread of disease. The purpose of this retrospective study was to determine the false negativity rate of the LumiraDx SARS-CoV-2 Ag Test when utilized for testing individuals suspected of SARS-CoV-2 infection.

**Methods:**

Concurrent swab samples were collected from patients suspected of SARS-CoV-2 infection by their healthcare provider within two different urgent care centers located in Easton, MA, USA and East Bridgewater, MA, USA. One swab was tested using the LumiraDx SARS-CoV-2 Ag Test. Negative results in patients considered at moderate to high risk of SARS-CoV-2 infection were confirmed at a regional reference laboratory by polymerase chain reaction (PCR) using the additional swab sample. The data included in this study was collected retrospectively as an analysis of routine clinical practice.

**Results:**

From October 19, 2020 to January 3, 2021, a total of 2241 tests were performed using the LumiraDx SARS-CoV-2 Ag Test, with 549 (24.5%) testing positive and 1692 (75.5%) testing negative. A subset (800) of the samples rendering a negative LumiraDx SARS-CoV-2 Ag Test was also tested using a PCR-based test for SARS-CoV-2. Of this subset, 770 (96.3%) tested negative, and 30 (3.8%) tested positive. Negative results obtained with the LumiraDx SARS-CoV-2 Ag test demonstrated 96.3% agreement with PCR-based tests (*CI* 95%, 94.7–97.4%). A cycle threshold (*C*_*T*_) was available for 17 of the 30 specimens that yielded discordant results, with an average *C*_*T*_ value of 31.2, an SD of 3.0, and a range of 25.2–36.3. *C*_*T*_ was > 30.0 in 11/17 specimens (64.7%).

**Conclusions:**

This study demonstrates that the LumiraDx SARS-CoV-2 Ag Test had a low false-negative rate of 3.8% when used in a community-based setting.

## Background

In December 2019, bronchoalveolar lavage specimens were collected from patients with pneumonia of unknown etiology in Wuhan, China [1]. Specimens were shown to be positive for the presence of a beta-coronavirus [1]. Whole genome sequencing analysis demonstrated that the viral isolates from 104 different patients showed a sequence homology of 99.9%, suggesting a common disease etiology [1]. Full-length genome sequencing showed that the novel viral isolate had a 96% sequence homology to a bat severe acute respiratory syndrome (SARS)-like coronavirus (CoV) strain called BatCoV RaTG13 [1]. The novel virus was named SARS coronavirus 2 (SARS-CoV-2), and the associated disease was named coronavirus disease 2019 (COVID-19) [2]. By March 2020, cases of COVID-19 had been identified in over 100 countries, and on March 11, 2020, the World Health Organization (WHO) declared a pandemic [3].

During the first two quarters of 2020, numerous SARS-CoV-2 diagnostic products entered the US market through the US Food and Drug Administration (FDA) emergency use authorization (EUA) program. As of June 2021, 28 different EUA-labeled rapid tests that identify the presence of SARS-CoV-2 antigen are available on the US market, most of which are performed at the point of care with results being available in less than 30 min with some tests requiring only 15 min or less to render a result [4]. In contrast, conventional molecular polymerase chain reaction (PCR)–based methods that are performed in the central diagnostic testing laboratory often take several hours to generate a result and 24–48 h to provide a result back to the patient. In many use cases, rapid SARS-CoV-2 antigen tests can provide a result before the patient even leaves the medical institution at which the test has been administered. The sooner infected individuals are identified, the sooner quarantine and contact tracing can be initiated. Thus, providing a rapid result to identify SARS-CoV-2–infected individuals can help to prevent and truncate the spread of disease [5–7].

Many available rapid SARS-CoV-2 antigen tests use lateral flow technology that has been shown to demonstrate poor sensitivity compared with PCR [8–11] with a recent pre-print study reporting that most tests demonstrate less than 40% sensitivity vs PCR in specimens with CT > 30 [12]. The rendering of a false-negative result to an individual delays or results in the failure to initiate quarantine and contact tracing. As a consequence of a false-negative result, many individuals with mild disease may believe they are not infected and may never return for another test and, thus, may contribute to the further transmission of disease throughout their disease course.

The LumiraDx SARS-CoV-2 Ag Test (LumiraDx UK Ltd; Alloa, UK) is a rapid microfluidic immunoassay that initially received EUA in August 2020 and identifies the presence of the SARS-CoV-2 nucleocapsid antigen in nasal swabs collected from individuals suspected of COVID-19 by their healthcare provider within 12 days of symptom onset [13]. The test renders a qualitative, digitally displayed positive or negative result within 12 min [14, 15]. The test is intended for use by laboratories certified under the CLIA that meet the requirements to perform moderate, high, or waived complexity tests [13]. This test is also authorized for use at the Point of Care (POC) (i.e., in patient care settings operating at least under a CLIA Certificate of Waiver) [13]. Waived tests are simple laboratory tests that have an insignificant risk of an erroneous result [16]. The results of a 12-item questionnaire evaluating the tests usability and safety conducted among eight healthcare workers were consistent with a simple easy-to-use test when utilized by at least minimally trained healthcare workers [15].

The clinical trial data summarized in the LumiraDx SARS CoV-2 antigen product insert states a product sensitivity of 97.6% in symptomatic individuals tested within 12 days of symptom onset [13]. These data demonstrated that the LumiraDx SARS CoV-2 antigen assay had a low false negativity rate of 1.2% in the 170 patients that tested negative with the LumiraDx SARS CoV-2 antigen test [13]. In this study, we sought to determine the false negativity rate in an expanded sample set and in a real-world, community-based healthcare setting. We were most interested in evaluating the false negativity rate since false-negative results can have significant public health consequences as individuals who believe they are not infected may be less inclined to engage in disease mitigation measures and thus may contribute to the further transmission of disease. As such, the results of this study were used to help determine the impact of introducing the test into a community-based healthcare setting. In this retrospective study, we determined the false negativity rate of the rapid SARS-CoV-2 antigen test from LumiraDx in a cohort of 800 patients within a community-based healthcare setting, by comparing the LumiraDx antigen result with PCR.

## Methods

### SARS-CoV-2 patient testing protocol

**Fig. 1:**
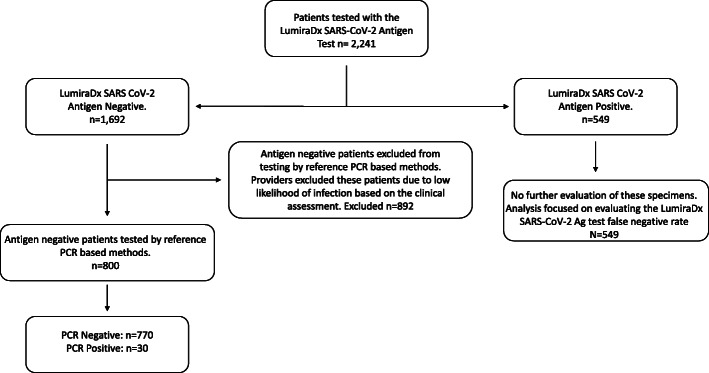
Study design and number of specimens tested with the LumiraDx SARS CoV-2 Ag Test and PCR-based methods. All symptomatic patients presenting at two different urgent care centers with 12 days of symptom onset were tested with the LumiraDx SARS-CoV-2 Antigen Test (*n* = 2241). A subset of these patients tested SARS CoV-2 Ag positive (*n* = 549) and negative (*n* = 1692). Based upon a healthcare provider’s assessment, patients with a low likelihood of SARS CoV-2 infection were excluded from further testing (*N* = 892). Patients with a moderate to high likelihood of SARS CoV-2 infection were also tested with a PCR-based method (*n* = 800). A subset of these patients tested positive (*n* = 30) and negative (*n* = 770) for SARS CoV-2 by PCR

All symptomatic patients presenting within 12 days of symptom onset were tested with the LumiraDx SARS-CoV-2 Ag Test. A small group of additional patients including eight patients that were > 12 days of symptom onset, three patients where symptom onset was not known, and 25 patients that were asymptomatic at the time of testing but had a documented recent exposure to a person testing positive for SARS-CoV-2 were also included in the study. A symptomatic patient was defined as an individual having at least one sign or symptom from those listed in Table 1. A subset of patients testing negative with the LumiraDx SARS-CoV-2 Ag Test in routine clinical practice were also tested with a PCR-based test for SARS-CoV-2. That subset of patients was determined during the initial clinical and risk assessment. This risk assessment was conducted for each patient by a healthcare provider and included a physical examination for clinical signs and a patient interview to assess clinical symptoms and patient history. If the healthcare provider determined the patient had a moderate to high likelihood of SARS-CoV-2 infection, then two swabs were concurrently collected. Only one swab was collected from patients determined to be low risk for SARS CoV-2 infection, and these patients were only tested with the LumiraDx SARS CoV-2 Ag Test. When two swabs were collected, one swab was placed into each nostril concurrently. An anterior nares swab specimen was collected as directed in the manufacturer’s package insert. The swabs were then switched, and the same swabs were used to collect a specimen from the other nostril. In this way, both of the concurrently collected swabs were equivalent. One swab was then immediately extracted and tested using the LumiraDx SARS-CoV-2 Ag Test (as further described below). If the LumiraDx SARS-CoV-2 Ag Test was positive, the second swab was discarded. If the LumiraDx SARS-CoV-2 Ag Test was negative, the second swab was prepared and sent to a reference laboratory for PCR-based SARS-CoV-2 testing. Figure 1 illustrates the study design and number of specimens tested with the LumiraDx SARS CoV-2 Ag Test and PCR-based methods.

**Table 1:** Common signs and symptoms of COVID-19 [17].

**Common signs and symptoms of COVID-19**	
Fever or chills Cough Shortness of breath or difficulty breathing Fatigue Muscle or body aches Headache New loss of taste or smell Sore throat Congestion or runny nose Nausea or vomiting Diarrhea	

*COVID-19*, coronavirus disease 2019

### Specimen collection and preparation

The LumiraDx SARS-CoV-2 Ag Test procedure involved the use of a single swab (Puritan HydraFlock Sterile Standard Flock Swab (Puritan Medical Products Company LLC; Guilford, ME, USA), or SteriPack Sterile Polyester Spun Swab (SteriPack USA Ltd LLC; Lakeland, FL, USA)) to swab the anterior nares of both nostrils. Both swab types utilized in the study were validated by the manufacturer for use with the LumiraDx SARS CoV-2 Ag Test. The manufacturer maintains a list of swabs that have been validated for use with the LumiraDx SARS CoV-2 Test at https://www.lumiradx.com/assets/pdfs/covid-19-antigen-test/sars-cov-2-antigen-technical-bulletin-swabs/sars-cov-2-ag-technical-bulletin-swabs-us.pdf?v=7. Within 1 h of collection, the nasal swab specimen was placed into the provided vial containing extraction buffer and eluted for 10 s. The swab was then swirled along the inside walls of the extraction vial five times. The outside walls of the extraction vial were gently squeezed together as the swab was removed from the vial and discarded. The squeezing action was intended to facilitate the extraction of liquid from the swab. A provided dropper top was then affixed to the top of the extraction vial, and the vial was then gently inverted five times. Specimens were interrogated within 5 h of swab extraction.

### SARS-CoV-2 antigen testing

All SARS-CoV-2 antigen testing was conducted at the East Bridgewater, MA or Easton, MA urgent care centers or the clinical diagnostic laboratory at East Bridgewater, MA within the Compass Medical system. Compass Medical is a physician-owned and -directed medical organization providing care to patients of all ages at six different locations across southeastern Massachusetts, USA. Compass Medical is an affiliate of Steward Health Care System, the largest community care organization in New England, USA.

Specimen collection was conducted by a trained clinician using universal precautions and in accordance with the manufacturer’s instructions. The LumiraDx SARS-CoV-2 Ag Test is a single-use, rapid microfluidic immunofluorescence assay that is authorized for use under the FDA EUA. The assay’s intended use states that the test detects the presence of nucleocapsid protein antigen to SARS-CoV-2 directly from nasal swab samples collected from individuals suspected of COVID-19 within the first 12 days of symptom onset. The testing procedure begins by powering on the LumiraDx Instrument. The instrument will prompt to install the lot calibration file when inserting a new test strip lot. Once installed, the instrument will have all the information required to process the test, and any future tests from the same lot of test strips. The instrument will indicate on the touchscreen when to open the foil pouch containing a single reagent test strip. When indicated, the LumiraDx Test Strip is inserted into the LumiraDx Instrument. The instrument will indicate when it is ready for the sample to be applied. When indicated, a single drop from the extracted sample contained within the extraction vial is applied onto the test strip. This is done by gently pressing the sides of the extraction vial until one whole drop of liquid is visible and then allowing it to touch the sample application area indicated on the end of the inserted test strip. The affixed dropper top is calibrated to deliver the required sample volume. The sample is then drawn by capillary action into the test strip. When the sample is detected, the instrument will sound (if sounds are enabled) and a confirmation message will be displayed. The touchscreen of the LumiraDx Instrument will then request the user to immediately close the door. The test result is determined from the amount of fluorescence the instrument detects within the measurement zone of the test strip. The concentration of SARS-CoV-2 antigen in the specimen is proportional to the fluorescence detected. A qualitative result of either POSITIVE or NEGATIVE is displayed on the instrument touchscreen within 12 min from applying the sample to the test strip and starting the test.

### SARS-CoV-2 PCR-based testing

**Fig. 2:**
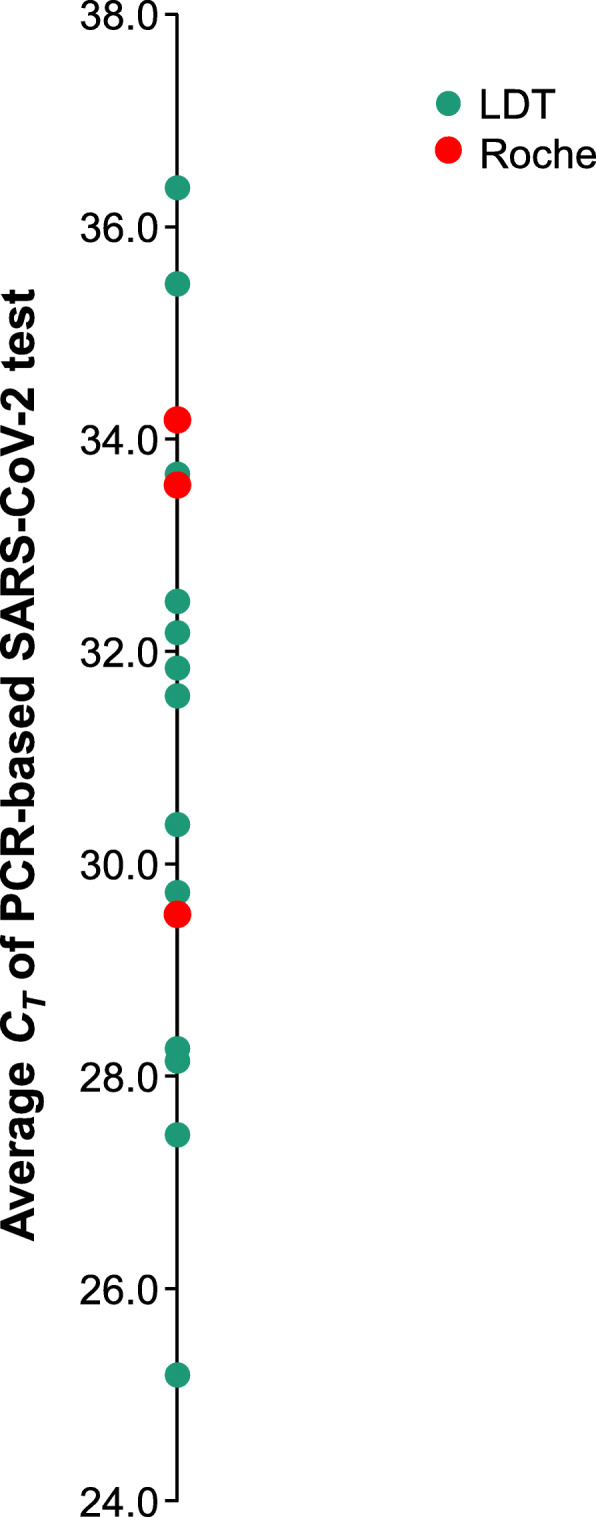
*C*_*T*_s were available for 56.8% of negative LumiraDx SARS-CoV-2 Ag Tests with positive PCR results. The average *C*_*T*_ between different SARS-CoV-2 gene and nucleic acid sequence targets is reported for each patient specimen represented. *C*_*T*_s were not available for SARS-CoV-2 testing performed using the Hologic method. *C*_*T*_, cycle threshold; (green circles) LDT, a laboratory-developed PCR testing method; *PCR*, polymerase chain reaction; (red circles) Roche PCR-based testing method; *SARS-CoV-2*, severe acute respiratory syndrome coronavirus 2

PCR-based testing for SARS-CoV-2 was performed at a national reference laboratory (Quest Diagnostics; Marlborough, MA, USA). Nasal swab specimens were collected as described above and in accordance with the recommendations provided in the online test catalogue. The reference laboratory utilized one of three PCR-based methods to conduct SARS-CoV-2 testing. Each of these methods targets different SARS-CoV-2 gene and nucleic acid sequences. Roche, Hologic, and a Quest Diagnostics LDT target SARS-CoV-2 open reading frame (ORF)-1a and E, ORF-1, and N1 and N3, respectively. CTs were obtained for all SARS-CoV-2 PCR-based testing performed using the Roche and LDT methods. CTs were not available for SARS-CoV-2 testing performed using the Hologic method. In this study, the average CT between different SARS-CoV-2 gene and nucleic acid sequence targets is reported for each patient specimen represented in Fig. 2.

### Ethical approval

This research was performed in accordance with Good Clinical Practice guidelines and the Declaration of Helsinki. Local ethical approval was obtained from Compass Medical Executive Board. The compass testing protocol was formulated independent of the study based on recommendations from the Compass Medical Chief Medical Officer and Urgent Care Physicians. The study represents a retrospective analysis of routine clinical practice. No additional specimens were collected in support of the study, and all analyses were conducted in a de-identified manner. As such, no patient informed consent was collected nor needed.

## Results

**Fig. 3:**
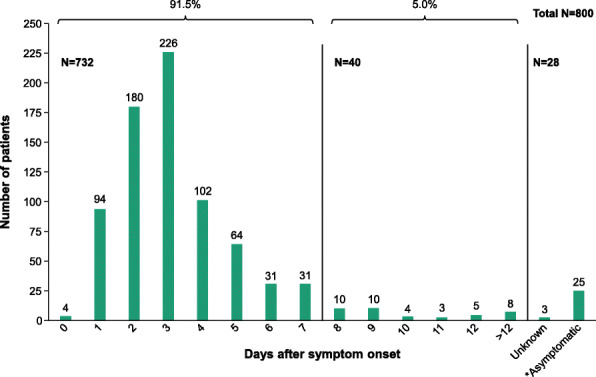
Days after symptom onset for patients tested with LumiraDx SARS-CoV-2 Ag Test and PCR-based method. There were 91.5% (732/800) of the patients tested within 7 days of symptom onset, while 5% (40/800) were tested more than 7 days after symptom onset. Number of days after symptom onset was not known in three patients, and 25* patients were asymptomatic at the time of testing but had a recent documented exposure to a person testing positive for SARS-CoV-2. All of the patients represented in the figure tested negative with the LumiraDx SARS CoV-2 Ag Test. All of the patients represented in the figure were also tested with a PCR based method, and 770/800 (96.3%) and 30/800 (3.8%) of these patients tested negative and positive, respectively. *PCR*, polymerase chain reaction; *SARS-CoV-2*, severe acute respiratory syndrome coronavirus 2

From October 19, 2020 to January 3, 2021, all symptomatic patients presenting within 12 days of symptom onset, at two different urgent care centers (East Bridgewater, MA or Easton, MA), within a community medical center setting were tested with the LumiraDx SARS-CoV-2 Ag Test. In addition, eight patients presenting > 12 days of symptom onset, three patients where the days of symptom onset was not known, and 25 asymptomatic patients but who had a documented recent exposure to a person testing positive for SARS-CoV-2 were also included in the analyses. During this time, a total of 2241 patients were tested, with 549 (24.5%) patients rendering a positive antigen result and 1692 (75.5%) rendering a negative antigen result (Table 2). During the initial clinical and risk assessment of the patients, 800 (47.3%) of the patients rendering a negative LumiraDx SARS-CoV-2 Ag Test result were determined to have moderate to high likelihood of SARS-CoV-2 infection, and thus were also tested using a PCR-based test for SARS-CoV-2 (Table 2). This cohort of patients comprised 337 (42.1%) and 463 (57.9%) males and females, respectively, and patients were distributed across six different age categories (Table 3). The distribution of this patient cohort in relationship to number of days after symptom onset showed that 732 (91.5%) of the patients were tested within 7 days after symptom onset, while 40 (5.0%) were tested more than 7 days after symptom onset (Fig. 3). The number of days after symptom onset was not known for three patients, and 25 patients were asymptomatic at the time of testing but had a documented recent exposure to a person testing positive for SARS-CoV-2 (Fig. 3). Overall, 775 (96.9%) of the patients included in the study were symptomatic at the time of testing. Of the 800 total patients tested, 770 (96.3%) of these patients tested negative, and 30 (3.8%) tested positive using a PCR-based test for SARS-CoV-2 (Table 2). This data demonstrates that negative results obtained with the LumiraDx SARS-CoV-2 Ag Test had 96.3% concordance (CI 95%, 94.7–97.4%) with PCR-based SARS-CoV-2 tests and a 3.8% false negativity rate (Table 2).

**Table 2:** SARS-CoV-2 test results from LumiraDx SARS-CoV-2 Ag Test and PCR-based test

**SARS CoV-2 rapid antigen tests**	
Total antigen tests conducted^a^ Positive antigen results rendered Negative antigen results rendered	2241 549 (24.5%) 1692 (75.5%)
**PCR-based SARS CoV-2 tests**	
Total PCR-based tests conducted^b^ Positive PCR results rendered Negative PCR results rendered	800 30 (3.8%) 770 (96.3%)

^a^All SARS-CoV-2 antigen testing (*n* = 2241) were conducted using the rapid, point-of-care LumiraDx SARS-CoV-2 Ag Test on patients presenting to an urgent care center

^b^During the initial clinical and risk assessment, a subset of patients (*n* = 800) that tested negative with the LumiraDx SARS-CoV-2 Ag Test was also tested with a PCR-based method for SARS-CoV-2

**Table 3:** Age and gender of patients tested with LumiraDx SARS-CoV-2 Ag Test and a PCR-based method

Gender	Patient age, ***n*** (%)						
	0–17	18–29	30–39	40–49	50–59	>60	Total
**Male** **Female**	18 (5.3) 16 (3.5)	98 (29.1) 34 (7.3)	56 (16.6) 84 (18.1)	43 (12.8) 117 (25.3)	58 (17.2) 118 (25.5)	64 (19.0) 64 (20.3)	337 (100) 463 (100)
							**800**

*SARS-CoV-2*, severe acute respiratory syndrome coronavirus 2.

*PCR*, polymerase chain reaction; *SARS-CoV-2*, severe acute respiratory syndrome coronavirus 2.

**Fig. 4:**
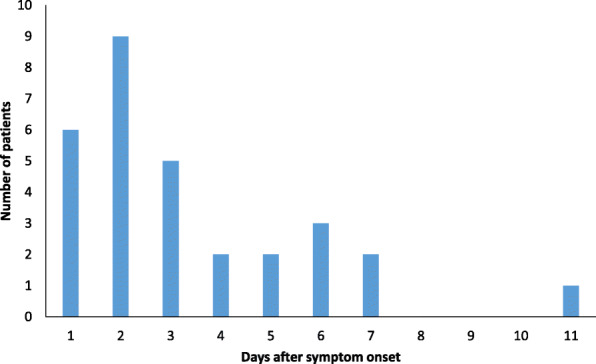
Distribution of days after symptom onset for patients testing negative with LumiraDx SARS-CoV-2 Ag Test and positive with a PCR-based method (*n* = 30). All patients were symptomatic and had an average number of days after symptom onset of 3.3 days. This population consisted of 13 males and 17 females and had an average age of 42.0 years

We further investigated the 30 specimens that rendered a negative LumiraDx SARS-CoV-2 Ag Test result and a positive PCR-based test result. Figure 4 shows the distribution of days after symptom onset for these patients. All patients were symptomatic and had an average number of days after symptom onset of 3.3 days. This population consisted of 13 males and 17 females and had an average age of 42.0 years. Cycle thresholds (C_T_s) were available for 17 of 30 (56.8%) of the specimens tested with a PCR-based testing method (Roche (Roche Cobas 6800 Platform, Roche Molecular Diagnostics, Indianapolis, IN, USA) and a laboratory-developed test (LDT)). C_T_s were not available for testing performed using the Aptima SARS-CoV-2 Assay (Hologic, Marlborough, MA, USA). The Roche and LDT PCR-based methods target different SARS-CoV-2 genes and nucleic acid sequences. The Roche method targets SARS-CoV-2 ORF-1a and E, while the LDT targets SARS-CoV-2 N1 and N3. In this analysis, the C_T_s for each target were averaged and reported for each patient, as represented in Fig. 2. The C_T_ average for all available C_T_s was 31.2 (*n* = 17), with a standard deviation (SD) of 3.0 and a range of 25.2–36.3. The majority (64.7%) of patients testing negative with the LumiraDx SARS-CoV-2 Ag Test and testing positive with a PCR-based test for SARS-CoV-2 had C_T_s > 30.0 (Fig. 2). The patients represented in Fig. 2 (*n* = 17) consisted of seven males and ten females, had an average age of 45.5 years, and averaged 3.4 days post symptom onset.

## Discussion

Providing a rapid result to identify SARS-CoV-2-infected individuals can help to prevent and truncate the spread of disease, as quarantine and contract-tracing measures can be initiated sooner than in individuals who do not receive such a test [5–7]. However, many of the available rapid SARS-CoV-2 antigen tests have been shown to lack sensitivity [8–11]. False-negative results can have significant public health consequences. In the context of a false-negative result, many individuals with mild disease may believe they are not infected and may never return for another test and, thus, may contribute to the further transmission of disease throughout their disease course. In this study, we show that the LumiraDx SARS-CoV-2 Ag Test has a low false negativity rate of 3.8% compared with PCR when used in a community-based healthcare setting.

As of October 15, 2021, 37 SARS CoV-2 Ag tests had received FDA EUA for use in the USA (4). However, a recent College of American Pathology SARS CoV-2 Ag proficiency testing report summary demonstrated that 689/1408 (89.1%) of laboratories reporting proficiency results for SARS CoV-2 Ag testing were utilizing either the BinaxNow (Abbott), Veritor (Becton Dickinson), or Sofia (Quidel) products [18]. The product package inserts for these leading rapid SARS-CoV-2 antigen tests report false-negative rates similar or even better than those reported for the LumiraDx SARS-CoV-2 Ag Test [19–21]. For example, the BinaxNOW COVID-19 Ag Card (Abbott Diagnostics Scarborough, Inc.; Scarborough, ME, USA) and the Veritor System for Rapid Detection of SARS-CoV-2 (Becton Dickinson and Company; Sparks, MD, USA) report false-negative rates of 5.1% and 2.5% respectively, while the Sofia 2 Flu + SARS Antigen FIA (Quidel Corporation; San Diego, CA, USA) reports a false-negative rate of only 0.6% [19–21]. However, the false negativity rate is influenced by disease prevalence, and hence false negativity rates may not be directly comparable, especially when disease prevalence varies significantly. In fact, the false negativity rates for these products were much higher when deployed and determined in an actual community setting [11, 22–24]. Furthermore, there are significant differences in the studies represented in the product package inserts compared with the study reported here. The studies represented in the product package inserts are all smaller than the study of 800 patients reported here, with the BinaxNOW COVID-19 Ag Card, Veritor System for Rapid Detection of SARS-CoV-2, and Sofia 2 Flu + SARS Antigen FIA studies consisting of 460, 226, and 164 patients, respectively [19–21]. Furthermore, the study of the Veritor System for Rapid Detection of SARS-CoV-2 only included symptomatic patients within 5 days of symptom onset [20]. The studies of the BinaxNOW COVID-19 Ag Card and Sofia 2 Flu + SARS Antigen FIA included symptomatic patients within 7 days of symptom onset, but the majority of patients included in the Sofia 2 Flu + SARS Antigen FIA study were within 4 days after symptom onset [19, 21]. The majority (95.5%, Fig. 3) of the patients included in the study reported here were symptomatic patients within 12 days of symptom onset. The LumiraDx SARS CoV-2 antigen assay product’s package insert reports a lower false negativity rate than demonstrated in this study, 1.2% [13] vs 3.8%, respectively. Only patients who had a moderate to high likelihood of SARS-CoV-2 infection (as determined during a physician-conducted clinical and risk assessment) were selected for concomitant LumiraDx and PCR testing, and thus, the false-negative rate reported here may be overestimated. A false-negative test result could be rendered when SARS-CoV-2 viral loads are low, such as very early [25, 26] and very late [27] in the disease course of COVID-19. Studies have demonstrated that rapid SARS-CoV-2 antigen tests have the greatest sensitivities when viral loads are high [28, 29]. This suggests that the false negativity rate of the BinaxNOW COVID-19 Ag Card, the Veritor System for Rapid Detection of SARS-CoV-2, and the Sofia 2 Flu + SARS Antigen FIA could have been higher if the respective studies had included patients who were tested more than 7 days of symptom onset, when viral load levels would have been declining. In support of this hypothesis, studies have demonstrated that the limit of detection for the Veritor System for Rapid Detection of SARS-CoV-2 and the Sofia 2 Flu + SARS Antigen FIA has a sensitivity equivalent to a molecular *C*_*T*_ of 27–28 [30]. In contrast, the LumiraDx SARS-CoV-2 Ag Test is more sensitive, with a sensitivity equivalent to a molecular *C*_*T*_ of < 33 [15]. The increased sensitivity of the LumiraDx SARS-CoV-2 Ag Test versus other rapid antigen tests is likely the consequence of the testing methodology. The LumiraDx SARS-CoV-2 Ag Test is a rapid microfluidic immunofluorescence assay that utilizes active control of the reaction time, volume, and temperature. The assay utilizes a wash step and a spectrophotometric read. In contrast, the BinaxNOW COVID-19 Ag Card, Veritor System for Rapid Detection of SARS-CoV-2, and Sofia 2 Flu + SARS Antigen FIA all utilize conventional lateral flow testing methodology. Lateral flow test methods entail only passive control of the specimen, with no wash steps.

The BinaxNOW COVID-19 Ag Card and the Sofia 2 Flu + SARS Antigen FIA both have FDA EUA claims for utilization in patients within up to 7 days of symptom onset, while the Veritor System for Rapid Detection of SARS-CoV-2 has an FDA EUA claim for utilization in patients within up to 5 days of symptom onset. Studies have demonstrated that SARS-CoV-2 can be successfully cultured in symptomatic patients 8–15 days following symptom onset [31–33]. If the presence of culturable virus provides at least some measure of infectivity, then it would be important that a rapid SARS-CoV-2 antigen test be able to identify infected patients more than 7 days after symptom onset. The LumiraDx SARS-CoV-2 Ag Test has an FDA EUA claim for utilization in patients up to 12 days after symptom onset, and this study demonstrated that the LumiraDx SARS-CoV-2 Ag Test has a low false-negative rate of 3.8% compared with PCR, even when patients presenting up to 12 days (Fig. 3, *n* = 764) and greater (*Fig.* *3*, *n* = 8) after symptom onset were included in the analysis.

This study also demonstrated that 11/17 (64.7%) of the false-negative results rendered by the LumiraDx SARS-CoV-2 Ag Test had *C*_*T*_s > 30. This suggests that many of the specimens rendering a false-negative result with the LumiraDx SARS-CoV-2 Ag Test had low viral loads and were potentially noninfectious [15]. A previous study demonstrated that the LumiraDx SARS-CoV-2 Ag Test had 100% concordance with SARS-CoV-2 PCR-positive specimens with *C*_*T*_s < 33 for specimens tested within 12 days post symptom onset [15]. The difference between the results of this previous study and those reported here may be reflective of the differences in the PCR assays used in each study. Drain et al. utilized the Roche SARS-CoV-2 PCR assay, while the study presented here primarily utilized an LDT SARS-CoV-2 PCR assay [15]. *C*_*T*_s achieved across different PCR-based assays are not directly comparable.

It should also be noted that this study was conducted from October 19, 2020 to January 3, 2021. The CDC did not start to list and categorize SARS CoV-2 variants until December, 2020 [34]. As such, the SARS CoV-2 alpha variant was likely to be the dominant SARS CoV-2 isolate during the time of this study. Further studies are needed to evaluate if other SARS CoV-2 isolates modulate the performance of the LumiraDx SARS CoV-2 antigen assay.

## Conclusions

Additional studies are needed to better characterize when individuals with SARS-CoV-2 infection are infectious and thus capable of transmitting disease. The development and optimization of the clinical and analytic performance of diagnostic tests to identify infectious individuals is of utmost importance to public health. This study demonstrated a low false-negative rate when using the LumiraDx SARS-CoV-2 Ag Test in a community-based setting.

## Data Availability

The datasets analyzed during the current study are available from the corresponding author on reasonable request.

## Abbreviations

Ag: Antigen
COVID-19: Coronavirus disease 2019
CT: Cycle threshold
EUA: US Food and Drug Administration (FDA) emergency use authorization (EUA) program
FDA: US Food and Drug Administration
LDT: Lab-developed test
ORF: Open reading frame
PCR: Polymerase chain reaction
RT-PCR: Real-time reverse transcription PCR
SARS-CoV-2: Severe acute respiratory syndrome (SARS)-like coronavirus (CoV) 2
SD: Standard deviation
WHO: World Health Organization
